# Discovery of Two GSK3β Inhibitors from *Sophora flavescens* Ait. using Structure-based Virtual Screening and Bioactivity Evaluation

**DOI:** 10.2174/0115734099321878241011104241

**Published:** 2024-10-25

**Authors:** Dabo Pan, Yong Zeng, Dewen Jiang, Yonghao Zhang, Mingkai Wu, Yaxuan Huang, Minzhen Han, Xiaojie Jin

**Affiliations:** 1 Department of Medical Technology, Qiandongnan Vocational and Technical College for Nationalities, Kaili, 556000, China;; 2 Department of Pharmacy, The Second Affiliated Hospital of Guizhou Medical University, Guizhou Medical University, Kaili, 556000, China;; 3 College of Pharmacy, The Affiliated Dazu’s Hospital of Chongqing Medical University, Dazu, 402360, China;; 4 College of Pharmacy, Gansu University of Chinese Medicine, Lanzhou, 730000, China

**Keywords:** GSK3β, Kushen, kushenol I, kushenol F, structure-based virtual screening, TMSCP database

## Abstract

**Objective:**

Kushen (*Sophora flavescens* Ait.) has a long history of medicinal use in China due to its medicinal values, such as antibacterial, antiviral, and anti-inflammatory. Rapid discovery of the components and the medicinal effects exerted by Kushen will help elucidate the science of Kushen in curing diseases. GSK3β (glycogen synthase kinase-3 beta) is a protein kinase with a wide range of physiological functions, such as antibacterial, antiviral, and anti-inflammatory. The discovery of inhibitors targeting GSK3β from Kushen was not only helpful for the rapid discovery of the components responsible for the efficacy of Kushen but also important for the development of novel drugs.

**Methods:**

In this study, the chemical composition of Kushen was extracted from the TMSCP database. Molecular docking, GSK3β enzyme assay, and molecular dynamics simulations were used to discover the GSK3β inhibitors from the chemical composition of Kushen.

**Results and Discussion:**

A total of 113 chemical compositions of Kushen were extracted from the TMSCP database. Molecular docking indicated that 15 chemical compositions of Kushen scored better than -8 kcal/mol against GSK3β. GSK3β enzyme assay demonstrated several inhibitory activities of kushenol I and kushenol F with IC_50_ values of 7.53 ± 2.55 µM and 4.96 ± 1.29 µM, respectively. Molecular dynamics simulations were used to reveal the interactions of kushenol I and kushenol F with GSK3β from structural and energetic perspectives.

**Conclusion:**

Kushenol I and kushenol F could be the material basis for the antibacterial, antiviral, and anti-inflammatory properties of Kushen.

## 1 INTRODUCTION

*Sophora flavescens* Ait., also known in Chinese as Kushen, has been widely used in the world for thousands of years. In traditional medicine, Kushen is frequently used to treat dysentery, intestinal catarrh, and eczema [1-3]. In the 2020 edition of the Chinese Pharmacopoeia, a variety of Chinese medicine formulas are mentioned, with Kushen as the main flavor, including Kushen tablets, Kushen capsules, Sophora ointment, *etc*. Currently, pharmacological studies have investigated that Kushen has antibacterial, anti-inflammatory, antiviral, antineoplastic, anticancer, antiarrhythmic, and antidiabetic properties [3-8]. Kushen is a phytopharmaceutical that is very rich in chemical constituents. Over 300 bioactive constituents have been isolated from kushen, including alkaloids, flavonoids, terpenoids, volatile oils, and fatty acids [4, 9, 10]. A large number of researchers have tried to explore the chemical constituents in Kushen that exert medicinal effects [4, 5]. The pharmacological studies on Kushen have mainly focused on alkaloids and flavonoids. Unfortunately, it is difficult to discover which chemical component in Kushen is the bioactive compound and how it works.

Currently, there are several strategies to find active compounds from structurally diverse natural chemicals, such as ligand-based target prediction, receptor-based target prediction, and artificial intelligence-based target prediction for computer-aided drug design and proteomics, genomics, and metabolomics for experimental assays [11-20]. Among these methods, integrating structure-based target prediction and enzyme bioactivity evaluation is an effective strategy for rapid target discovery [21-29]. It is helpful in discovering potential targets for compounds by purposeful search for potential docking targets.

GSK3β slows the conversion of glucose to glycogen by weakening the catalytic activity of the substrate (glycogen synthase) through phosphorylation and has become an attractive target for the treatment of type 2 diabetes [30]. Furthermore, GSK3β plays a primary role in the Wnt/β-catenin pathway that is involved in lung diseases, heart diseases, liver diseases, hair disorders, bone diseases, and nervous system diseases [31]. In addition, the central role of GSK3β is in the regulation of pathogen-induced inflammatory responses through the regulation of pro- and anti-inflammatory cytokine production. In summary, GSK3β is a crucial regulatory kinase interacting with multiple pathways to control various physiological processes, as well as the treatment of various human diseases, including diabetes, neurodegenerative disease, mood disorders, inflammation, cancer, and autophagy with several antibacterial, anti-inflammatory, antiviral, antineoplastic, antiarrhythmic, and antidiabetic properties [32-39]. The discovery of inhibitors targeting GSK3 from Kushen has helped us to find the material basis of antibacterial, anti-inflammatory or anti-tumor properties of Kushen.

In this study, the bioactive constituents of Kushen were extracted from an open-source database of chemical constituents of traditional Chinese medicine, and then the GSK3β candidates (kushenol I and kushenol F) were screened from the bioactive constituents of Kushen based on structure-based target prediction and enzyme activity test. Molecular dynamics and MM/GBSA approaches were integrated to explore the mechanism of inhibitors-GSK3β interaction from energetic and structural perspectives. This was conducive to the elucidation of the material basis and mechanism of action of Kushen.

## 2 MATERIALS AND METHODS

### 2.1 Collection of Chemical Compositions in Kushen

In the Traditional Chinese Medicine Systems Pharmacology Database and Analysis Platform (TCMSP) [40], the chemical compositions of Kushen were screened using the herb name *Sophorae Flavescentis* Ait., and their 3D structures were downloaded from TCMSP. These structures were added by energy minimization using the LigPrep module [41] in Schrödinger 2017 [42] using the OPLS-2005 force field. The possible partial charges were generated at pH 7 ± 2 using the Epik module [43]. Each structure remained specified chirality and generated at most 32 conformations.

### 2.2 Structure-based Virtual Screening

The extra precision (XP) Glide module [44] was employed to dock the chemical compositions of Kushen to the GSK3β kinase. The 3D structure of GSK3β was extracted from the Protein Data Bank (PDB ID: 4AFJ) [45]. Chain A remained, and then all waters, SO_4_^2-^, and GOL were deleted. The missing loops were added using Protein Preparation Wizard. The center and size of the binding site grid were identified according to the centroid ligand position in the GSK3β-ligand complex (PDB ID: 4AFJ; and 20 Å × 20 Å × 20 Å, respectively). Furthermore, the binding site grid was generated by the Receptor Grid Generation module, including Ile62, Tyr134, Val135, and Pro136. Detailed molecular docking was performed according to the methods described previously [46]. In molecular docking, 100 poses were generated during the initial phase of the docking calculation, out of which the best 10 poses were chosen for energy minimization by 1000 steps of conjugate gradient minimizations.

### 2.3 *In vitro* GSK3β Enzymatic Assay

The *in vitro* GSK3β enzymatic assay was performed as previously described [47, 48]. The enzymatic reactions were carried out at 30°C for 40 minutes. The 50µl reaction mixture consisted of 40 mM Tris, pH 7.4, 10 mM MgCl_2_, 0.1 mg/ml BSA, 1 mM DTT, 10 µM ATP, kinase, and the enzyme substrate. This was done to ensure that the final concentration of dimethyl sulphoxide in all reactions was 1%. Afterward, the compound was diluted to be tested with 10% dimethylsulfoxide, and then 5µl of the diluent was added to 50µl of the reaction. The Kinase-Glo Plus Luminous Kinase Assay Kit was used for the assay. The amount of ATP remaining in the solution after the kinase reaction was measured to determine kinase activity. The IC_50_ values of the compounds for inhibition of enzyme activity were calculated by normalised dose-response fitting non-linear regression using Prism GraphPad software.

### 2.4 Molecular Dynamics Simulation and MM/GSA Free Energy Calculation

The parameters of the small molecules were generated using GAFF of the AMBER force field, and the charges of the small molecules were obtained by Gaussian09 optimized at the HF/6-31G* level using RESP fitting [49]. Amber20 software was used to perform molecular dynamics simulations of all systems [50]. The AMBER14 force field [51] and TIP3P water [52] were used for proteins and water molecules, respectively. To ensure that the total charge of the system was 0, the counterion Cl- was added for neutralization. A 10 Å water molecule box was added for dissolution. In order to explore the interaction model between ligand and GSK3β, three systems, including GSK3β-Kushenol I complex, GSK3β-kushenol F complex, and GSK3β-5-(4-methoxyphenyl)-N-(pyridin-4-ylmethyl)-1, 3-oxazole-4-carboxamide complex (GSK3β-SJJ complex) [45], were constructed and simulated in parallel three times for 200 ns. Detailed molecular dynamics simulations were performed as described previously [53-57]. The system was heated from 0 K to 310 K after 5000 steps of energy minimization. Finally, 200 ns MD simulations at a temperature of 310 K and pressure of 1 atm were carried out without any restraint. In order to obtain the free energy of binding between the ligand and GSK3β, the molecular mechanics generalized Born surface area (MM/GBSA) method [58] was used to calculate their binding free energies. One snapshot of the structure was taken every 100 ps for the calculation of the binding free energy, and 50 frames of the structure were used for the entropy calculation. Each snapshot was optimized for 100 000 steps until the RMSD of the gradient vector was less than 0.0001 kcal/mol•Å^2^. In order to identify the key amino acids between the ligand and GSK3β, the binding free energy was decomposed to each amino acid and the amino acids with an energy contribution value greater than -1 kcal/mol were taken as key amino acids.

## 3 RESULTS AND DISCUSSION

### 3.1 The Chemical Composition of Kushen

One hundred thirteen chemical compositions of Kushen were extracted from TCMSP and are mentioned in Table **S1**. These chemical compositions contained alkaloids, flavonoids, terpenoids, volatile oils, and fatty acid. The basic skeleton of flavonoids in Kushen mainly included dihydroflavonoids, dihydroflavonols, isoflavonoids, flavonoids, chalcones, and zanthoxanes. The representative dihydroflavonoids contained MOL006625 (leachianone A) and MOL006641 (sophoraflavone G). The representative dihydroflavonols were composed of MOL006617 (kushenol F) and MOL006585 (kushenol I). The alkaloids were another major group in Kushen, mainly including MOL005944 (matrine) and MOL006634 (oxymatrine).

### 3.2 The Candidate Compounds by Virtual Screening

In order to assess the reliability of molecular docking in the extra precision (XP) Glide module, the ligand, namely 5-(4-methoxyphenyl)-N-(pyridin-4-ylmethyl)-1,3-oxazole-4-carboxamide (SJJ), in GSK3β complex was redocked into the binding site of GSK3β (PDB ID: 4AFJ), and the RMSD of the redocked SJJ stacked with the ligand in the crystal was 0.75 Å, less than 1Å, indicating that the molecular docking method used in this study was credible. The docking score between SJJ and GSK3β was -6.860 kcal/mol, which suggested that the ligand with a docking score of less than -6.860 kcal/mol was a potential GSK3β inhibitor [59]. Furthermore, the top 15 chemical compositions of Kushen that scored better than -6.860 kcal/mol against GSK3β were chosen for further experiments (Table **1**). Among the chemical compositions of Kushen, the docking score between MOL006619 (kushenol J) and GSK3β was the best, *i.e*., -11.777 kcal/mol. Compared with kushenol J, MOL000009 (luteolin-7-o-glucoside) was weakly bound to GSK3β with -11.154 kcal/mol. Due to the small stock of compounds and their high price, only MOL006585 (kushenol I) and MOL006617 (kushenol F) (Fig. **S1**) were purchased, and their GSK3β enzymatic activity was tested in the next step.

### 3.3 Kushenol I and kushenol F Inhibited GSK3β Enzymatic Activity

**Fig. (1):**
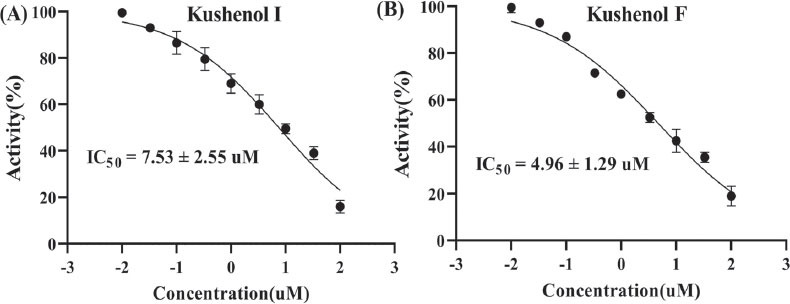
Dose-response curves of two GSK3β inhibitors: (**A**) kushenol I; (**B**) kushenol F.

In order to determine whether the compounds were active against GSK3β, we tested the activity of the compounds to inhibit enzyme GSK3β at a concentration of 30 µM. Kushenol I and kushenol F reduced GSK3β activity by 59% and 63% at a concentration of 30 µM, respectively. This indicated that kushenol I and kushenol F showed relative strong inhibitory activity. Furthermore, the IC_50_ values of the kushenol I and kushenol F against GSK3β were tested. Interestingly, kushenol I and kushenol F inhibited GSK3β activity in a concentration-dependent manner. The IC_50_ values of kushenol I and kushenol F were 7.53 ± 2.55 µM and 4.96 ± 1.29 µM, respectively (Fig. **1**).

### 3.4 Interaction Mechanism between Candidates and GSK3β

**Fig. (2):**
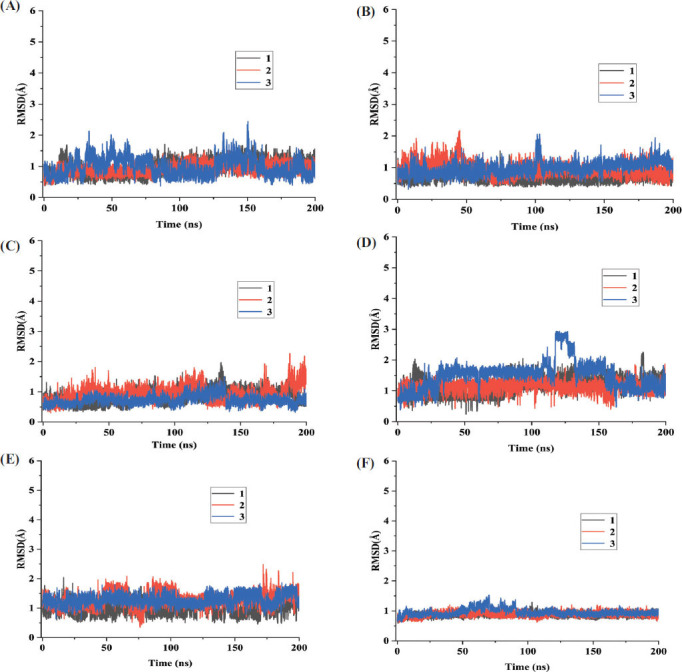
The monitoring of RMSDs in parallel three times: time evolution of RMSD of Cα atoms for the residues around 5 Å of ligands, (**A**) the GSK3β-kushenol I complex; (**B**) the GSK3β-kushenol F complex, (**C**) the GSK3β-SJJ complex; time evolution of the RMSD of heavy atoms of ligand, (**D**) kushenol I, (**E**) kushenol F, (**F**) SJJ.

**Fig. (3):**
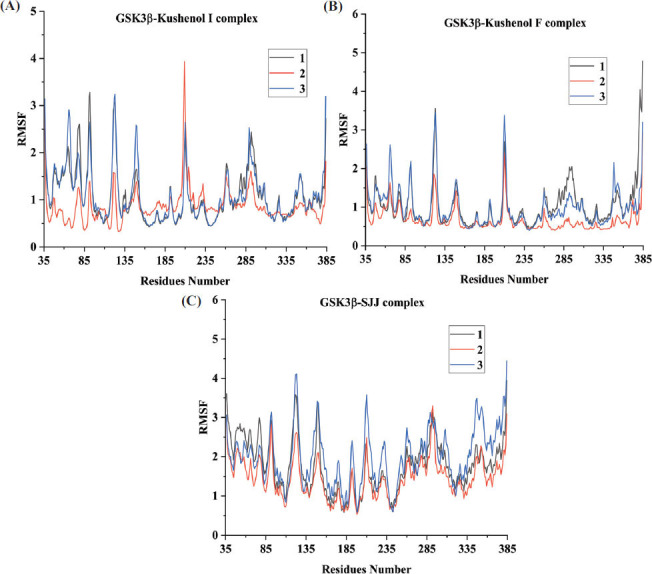
The RMSFs relative to the initial structure for backbone atoms of the GSK3β, (**A**) the GSK3β-kushenol I complex; (**B**) the GSK3β-kushenol F complex, (**C**) the GSK3β-SJJ complex.

In order to explore the detailed interaction between candidates and GSK3β, molecular dynamics simulation, binding free energy calculation, and binding free energy decomposition were employed. The RMSDs (Root mean square deviations) of the binding site (the residues around 5 Å of ligands) and heavy atoms of ligands were monitored and are shown in Fig. (**2**). It was observed that each system had a tiny fluctuation from 0.5 to 2.5 Å, indicating that each system achieved equilibrium in all molecular dynamics simulations. The trend of the RMSD of the three trajectories was consistent. The RMSD of the heavy atoms of ligands of the GSK3β-SJJ complex exhibited the least fluctuation among the three systems, with a range of 0.5 to 1.5 (Fig. **2F**). Interestingly, the RMSDs of the active site and heavy atoms of ligands of the GSK3β-kushenol I complex (Figs. **2A** and **2D**) were relatively larger than those of the GSK3β-kushenol F complex (Figs. **2B** and **2E**). The larger fluctuation of kushenol F in GSK3β may be caused by the poorer inhibitory activity. Furthermore, the RMSFs relative to the initial structure for backbone atoms of the GSK3β among the three systems were monitored and are shown in Fig. (**3**). Amino acid fluctuations were low in all regions except the N-terminal, C-terminal, and loop region of GSK3β (Fig. **3**).

The calculated binding free energies of the three parallel trajectories for each system were different, but the binding affinities of the three systems are ordered as follows: the GSK3β-SJJ complex > the GSK3β-kushenol F complex > the GSK3β-kushenol I complex, which was consistent with the results of the enzymatic GSK3β activity (Table **2**). For convenience, we selected the results of the first trajectory for the later analysis. Among three systems, the calculated binding free energy of the GSK3β-SJJ complex, the GSK3β-kushenol F complex, and the GSK3β-kushenol I complex were -10.01 kcal/mol, -7.90 kcal/mol, and -5.40 kcal/mol, respectively. From the energy terms, the nonpolar energy term (ΔG_nonpolar_) provided the main driving force for the binding of candidates, while both the polar energy term (ΔG_polar_) and the entropy term (-TΔS) were unfavorable to inhibitor binding. In GSK3β- kushenol I complex, the nonpolar energy term and the polar energy term were -50.97 kcal/mol and 25.25 kcal/mol, respectively. The GSK3β-kushenol F complex and the GSK3β-kushenol I complex exhibited superior nonpolar interaction terms in comparison to the GSK3β-SJJ complex. In contrast, the polar interaction terms and entropy penalties were also higher, resulting in a lower binding affinity for the GSK3β-kushenol F complex and the GSK3β-kushenol I complex in comparison to the GSK3β-SJJ complex.

To disclose the detailed interactions between inhibitors and GSK3β, the per-residue contribution profiles for the binding of inhibitors are plotted in Fig. (**4**). In GSK3β- kushenol I complex, there were seven amino acids, including Ile62, Phe67, Val70, Gln185, Asn186, Leu188, and Cys199, with more than 1 kcal/mol energy contribution. Compared with the GSK3β- kushenol I complex, two amino acids, including Leu132 and Tyr134, were favorable to the binding of GSK3β and kushenol F with an energy contribution of more than 1 kcal/mol, while the energy contribution terms of Gln185 and Asn186 were lower. Although the key amino acids were different in each system, the energy contributions of amino acids, Ile62, Val70, and Leu188, were greater than -1 kcal/mol in all three systems. Moreover, the representative snapshots were taken from the simulation trajectory and are shown in Fig. (**5**).

**Fig. (5):**
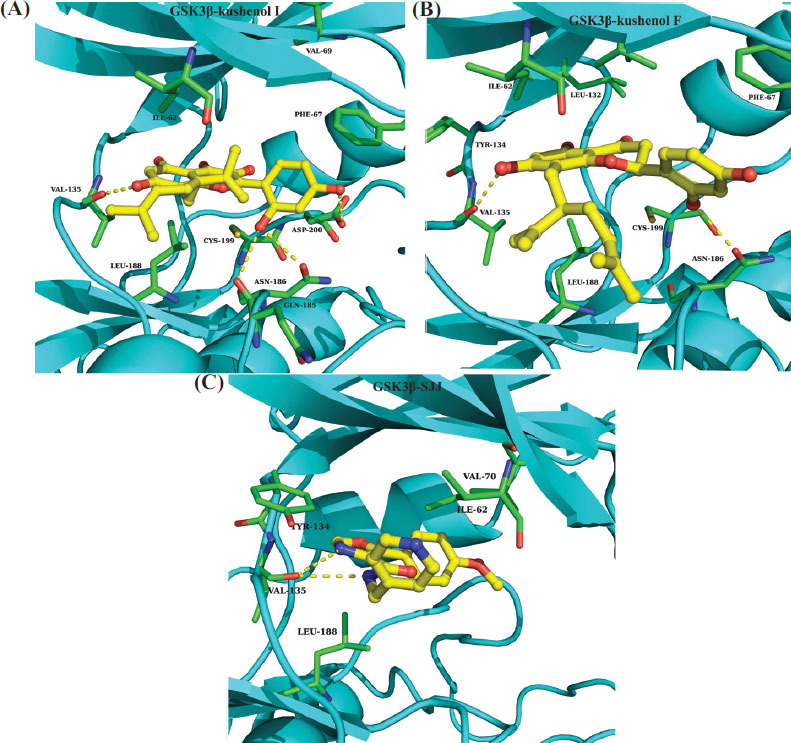
The interactions between candidates and GSK3β: (**A**) the GSK3β-kushenol I complex; (**B**) the GSK3β-kushenol F complex, (**C**) the GSK3β-SJJ complex.

As shown in Fig. (**5**), kushenol I was well-embedded in the active pocket of GSK3β, forming one hydrogen bond with the main chain of Val135 and two hydrogen bonds with the side chains of Asn186 and Lys85. Furthermore, there was a strong π-π stacking between kushenol I and Phe67 (Fig. **5A**). Related to the GSK3β-kushenol I complex, the hydrogen bond between the ligand and the hinge region (Val135) was still preserved in the GSK3β-kushenol F complex, which was monitored in many kinase inhibitors of GSK3B and was a significant feature of GSK3B inhibitors [60-68]. The presence of a hydroxyl-containing hydrophilic side chain of kushenol F made it more favorable to interact with the solvent around the active pocket, which could be the main reason why kushenol F bound better to GSK3B than kushenol I (Fig. **5B**). In the GSK3β-SJJ complex, there were two hydrogen bonds between SJJ and the hinge region (Val135). In order to reveal the crucial hydrogen bond between the inhibitor and GSK3β interaction, the hydrogen bonds between them were assessed and are mentioned in Table **3**. The main chain of Val135 in the hinge region formed a strong hydrogen bond with three inhibitors with more than 54% occupation in three systems. Meanwhile, the hydrogen bond between Asn186 and two candidate compounds was relatively weak, *i.e*., less than 20% of the occupation.

Kushenol I and kushenol F were widely found in natural medicines, such as Kushen. Kushenol I displayed significant antibacterial activities against A. *baumannii* [6], *Staphylococcus aureus*, *Bacillus subtilis*, *S. epidermidis*, and *Propionibacterium acnes* [69]. In addition, kushenol F exerted modest antibacterial activity [70-72], antiviral activity [73], and anti-inflammatory effects [74, 75]. In this study, molecular simulation and GSK3β enzymatic assay indicated that these two candidate compounds, including kushenol I and kushenol F, were able to bind to GSK3β directly and exert antibacterial, antimicrobial, and anti-inflammatory effects. This indicated that kushenol I and kushenol F could be the material basis for the antibacterial, antiviral, and anti-inflammatory properties of of Kushen.

## STUDY LIMITATIONS

The integration of molecular modeling and enzyme testing facilitates the rapid discovery of chemical compounds targeting GSK3β. However, the difficulty in obtaining plant chemical constituents has made it challenging to experimentally validate certain chemical compounds that showed promising predictive results in molecular simulations. This imposes certain limitations on the elucidation of the material basis for medicinal plants in treating specific diseases.

## CONCLUSION

Integrating molecular docking, molecular dynamics simulation, and enzyme activity assay, we identified the inhibitors kushenol I and kushenol F from Kushen targeting GSK3β and elucidated the interaction mode of kushenol I and kushenol F with GSK3β. The ability of kushenol I and kushenol F to better inhibit GSK3B indicated that they could be the material basis for Kushen to exert antibacterial, antiviral, and anti-inflammatory effects. The integration of molecular docking, molecular dynamics simulation, and enzyme activity tests could be a rapid method to discover the material basis for the exertion of medicinal effects by traditional Chinese medicine.

## Figures and Tables

**Fig. (4):**
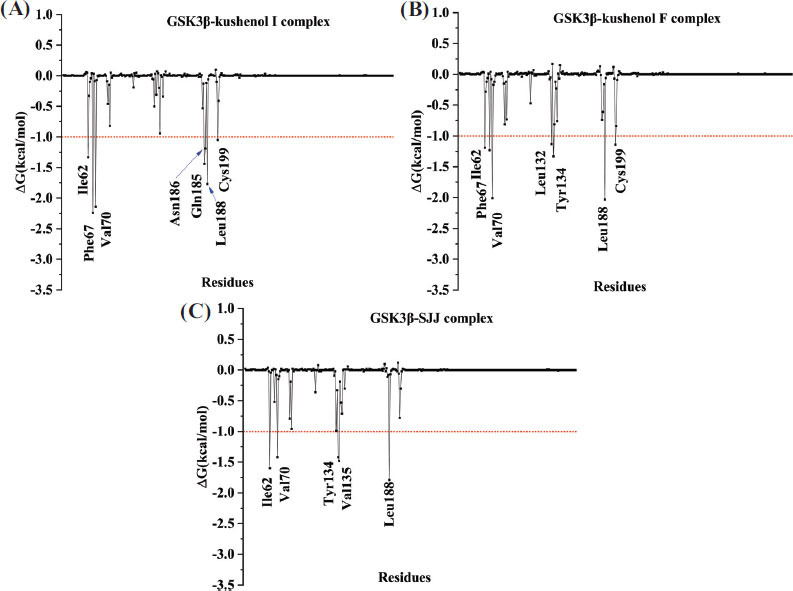
The residue contributions of GSK3β to candidate binding: (**A**) the GSK3β- kushenol I complex; (**B**) the GSK3β- kushenol F complex; (**C**) the GSK3β-SJJ complex.

**Table 1:** The docking score of the chemical composition of Kushen in GSK3β.

**No.**	**Title**	**Docking Score (kcal/mol)**	**Molecule Name**
1	MOL006619	-11.777	kushenol J
2	MOL000009	-11.154	Luteolin-7-o-glucoside
3	MOL000098	-9.096	quercetin
4	MOL004580	-9.076	cis-Dihydroquercetin
5	MOL006650	-8.966	(-)-Maackiain-3-O-glucosyl-6'-O-malonate
6	MOL006632	-8.812	(2S)-2-(3,4-dihydroxyphenyl)-6-[(2E)-3,7-dimethylocta-2,6-dienyl]-5,7-dihydroxychroman-4-one
7	MOL006618	-8.714	Kushenol G
8	MOL006641	-8.555	Sophoraflavanone,g
9	MOL003673	-8.507	Wighteone
10	MOL006630	-8.496	Norartocarpetin
11	MOL000006	-8.405	Luteolin
12	MOL006585	-8.401	Kushenol I
13	MOL006617	-8.239	Kushenol F
14	MOL003542	-8.094	8-Isopentenyl-kaempferol
15	MOL006626	-8.003	Leachianone g

**Table 2:** The calculated binding free energies of three parallel trajectories (kcal/mol).

**-**	**Kushenol I**			**Kushenol F**			**SJJ^c^**		
**-**	**n = 1**	**n = 2**	**n = 3**	**n = 1**	**n = 2**	**n = 3**	**n = 1**	**n = 2**	**n = 3**
ΔE_ele_	-21.54	-25.37	-24.37	-23.32	-22.59	-23.17	-23.12	-22.49	-19.35
ΔE_vdw_	-44.02	-42.37	-42.61	-44.13	-43.26	-44.01	-39.29	-39.71	-38.63
ΔG_sol-np_	-6.95	-6.68	-6.74	-6.61	-6.52	-6.58	-5.57	-5.57	-5.50
ΔG_sol-ele_	46.79	47.53	47.38	46.14	45.33	46.54	39.37	39.19	36.16
ΔG_nonpolar_^b^	-50.97	-49.05	-49.35	-50.74	-49.78	-50.59	-44.86	-45.28	-44.13
ΔG_polar _^a^	25.25	22.16	23.01	22.82	22.74	23.37	16.25	16.70	16.81
ΔH_bind_	-25.72	-26.89	-26.34	-27.92	-27.04	-27.22	-28.61	-28.58	-27.32
T∆S	-20.32	-20.84	-21.13	-20.02	-19.48	-19.94	-18.60	-18.83	-18.09
ΔG_bind_	-5.40	-6.05	-5.21	-7.90	-7.56	-7.28	-10.01	-9.75	-9.23
IC_50_ (µM)	7.53 ± 2.55			4.96 ± 1.29			0.40^d^		

**Note:** ^a^ ΔG_polar_ = ΔE_ele_ + ΔE_vdw_, ^b^ ΔG_nonpolar_ = ΔG_vdw_ + ΔG_sol-np_.^c^ 5-(4-methoxyphenyl)-N-(pyridin-4-ylmethyl)-1,3-oxazole-4-carboxamide, ^d^ ref 28.

**Table 3:** Percentage occupation of hydrogen bonds between GSK3β and inhibitors.

**Ligand**	**Acceptor**	**DonorH**	**Donor**	**Frames**	**Present (%)**	**Average Distance**	**Average Angle**
kushenol I	Asn186@OD1	MOL385@H21	MOL385@O6	3460	17.30%	2.72	160.11
	Asp200@OD1	MOL385@H22	MOL385@O7	6816	34.08%	2.63	162.68
	Asp200@OD2	MOL385@H22	MOL385@O7	4503	22.52%	2.63	162.54
	Asp200@OD1	MOL385@H21	MOL385@O6	3131	15.66%	2.67	157.36
	Asp200@OD2	MOL385@H21	MOL385@O6	2337	11.69%	2.69	155.68
	Val135@O	MOL385@H11	MOL385@O1	9318	46.59%	2.76	161.48
kushenol F	Asn186@OD1	MOL385@H31	MOL385@O7	3675	18.38%	2.75	158.57
	Val135@O	MOL385@H21	MOL385@O2	15475	77.38%	2.77	161.02
SJJ	Val135@O	MOL385@HN16	MOL385@N16	12508	62.54%	2.86	145.26

## Data Availability

All data generated or analysed during this study are included in this published article.

## LIST OF ABBREVIATIONS

GSK3β: Glycogen Synthase Kinase-3 Beta
MD: Molecular Dynamics Simulation
MM/GBSA: Molecular Mechanics Generalized Born Surface Area
RMSD: Root Mean Square Deviation
TCMSP: The Traditional Chinese Medicine Systems Pharmacology Database and Analysis Platform
