# Supplementary material for: Discovery of Two GSK3β Inhibitors from Sophora flavescens Ait. using Structure-based Virtual Screening and Bioactivity Evaluation
Source: Curr Comput Aided Drug Des. 2024 Oct 25;22(1):14–24. doi: 10.2174/0115734099321878241011104241 (PMC13358776; doi:10.2174/0115734099321878241011104241)
Supplement: Supplementary file 1 [file CCADD-22-1-14_SD1.pdf]

SUPPLEMENTARY MATERIAL

Discovery of Two GSK3β Inhibitors from *Sophora flavescens* Ait. using Structure-based Virtual Screening and Bioactivity Evaluation

Dabo Pan[1,2,*], Yong Zeng[3], Dewen Jiang[1], Yonghao Zhang[1], Mingkai Wu[1], Yaxuan Huang[1], Minzhen Han[2] and Xiaojie Jin[4,*]

[1]Department of Medical Technology, Qiandongnan Vocational and Technical College for Nationalities, Kaili, 556000, China; [2]Department of Pharmacy, the Second Affiliated Hospital of Guizhou Medical University, Guizhou Medical University, Kaili, 556000, China; [3]College of Pharmacy, the Affiliated Dazu's Hospital of Chongqing Medical University, Dazu, 402360, China; [4]College of Pharmacy, Gansu University of Chinese Medicine, Lanzhou, 730000, China

Table S1. 113 chemical composition of kushen were extracted from TMSCP.

No.	Mol ID	Molecule Name	No.	Mol ID	Molecule Name
1	MOL000006	luteolin	58	MOL006596	Glyceollin
2	MOL000008	apigenin	59	MOL006597	OMD
3	MOL000009	luteolin-7-o-glucoside	60	MOL006599	3,4',5-Trihydroxy-7-methoxy-8-isopente-nylflavone
4	MOL000098	quercetin	61	MOL006600	1-[2,4-dihydroxy-3-(3-methylbut-2-enyl)phenyl]-3-phenylprop-2-en-1-one
5	MOL000392	formononetin	62	MOL006601	kuraranine
6	MOL000393	Soyasaponin I	63	MOL006602	isokurarinone
7	MOL000456	Phaseolin	64	MOL006603	(E)-1-(2,6-dihydroxyphenyl)-3-(4-hydroxyphenyl)prop-2-en-1-one
8	MOL000657	Kushenol E	65	MOL006604	(2S)-7-hydroxy-2-(4-hydroxyphenyl)-5-methoxy-8-(3-methylbut-2-enyl)chroman-4-one
9	MOL001040	(2R)-5,7-dihydroxy-2-(4-hydroxyphenyl)chroman-4-one	66	MOL006605	kosamol,q
10	MOL001484	Inermine	67	MOL006606	kosamol,r
11	MOL003347	hyperforin	68	MOL006607	kurarainone
12	MOL003542	8-Isopentenyl-kaempferol	69	MOL006608	kuraridin
13	MOL003627	sophocarpine	70	MOL006609	kuraridine
14	MOL003641	Soyasapogenol B	71	MOL006610	kuraridinol
15	MOL003648	Inermin	72	MOL006611	kurarinol
16	MOL003673	Wighteone	73	MOL006612	kurarinone
17	MOL003676	Sophoramine	74	MOL006613	kushenin

1875-6697/26 $65.00+.00 © 2026 Bentham Science Publishers

No.	Mol ID	Molecule Name	No.	Mol ID	Molecule Name
18	MOL003680	sophoridine	75	MOL006614	kushenol A
19	MOL004580	cis-Dihydroquercetin	76	MOL006615	kushenol B
20	MOL004941	(2R)-7-hydroxy-2-(4-hydroxyphenyl)chroman-4-one	77	MOL006616	kushenol D
21	MOL005100	5,7-dihydroxy-2-(3-hydroxy-4-methoxyphenyl)chroman-4-one	78	MOL006617	kushenol F
22	MOL005944	matrine	79	MOL006618	KushenolG
23	MOL006561	(+)-14alpha-hydroxymatrine	80	MOL006619	kushenol J
24	MOL006562	(+)-7,11-dehydromatrine,(leontalbinine)	81	MOL006620	kushenol J_qt
25	MOL006563	(+)-9alpha-hydroxymatrine	82	MOL006621	(2R,3R)-2-(2,4-dihydroxyphenyl)-3,7-dihydroxy-8-[(2R)-2-isopropenyl-5-methylhex-4-enyl]-5-methoxy-4-chromanone
26	MOL006564	(+)-allomatrine	83	MOL006622	kushenol O
27	MOL006565	AIDS211310	84	MOL006623	kushenol,t
28	MOL006566	(+)-lehmannine	85	MOL006624	kushequinone A
29	MOL006567	(+)-sophoranol	86	MOL006625	leachianone,a
30	MOL006568	isosophocarpine	87	MOL006626	leachianone,g
31	MOL006569	(-)-14beta-hydroxymatrine	88	MOL006627	Lehmanine
32	MOL006570	(-)-9alpha-hydroxysophoramine	89	MOL006628	(+)-Lupanine
33	MOL006571	anagyrine	90	MOL006629	mamanine
34	MOL006572	1,4-diazaindan-type,alkaloid,flavascensine	91	MOL006630	Norartocarpetin
35	MOL006573	13,14-dehydrosophoridine	92	MOL006631	(2R)-2-(2,4-dihydroxyphenyl)-5,7-dihydroxy-8-[(2S)-2-isopropenyl-5-methylhex-4-enyl]-4-chromanone
36	MOL006574	2-Hydroxychalcone	93	MOL006632	(2S)-2-(3,4-dihydroxyphenyl)-6-[(2E)-3,7-dimethylocta-2,6-dienyl]-5,7-dihydroxychroman-4-one
37	MOL006575	2-n-hencosyl-5,7-dihydroxy-6,8-dimethylchromone	94	MOL006633	7-(3-methylbut-2-enoxy)-2-chromenone
38	MOL006576	2-n-heptadecyl-5,7-dihydroxy-6,8-dimethyl chromone	95	MOL006634	oxymatrine
39	MOL006577	2-n-nonadecyl-5,7-dihydroxy-6,8-dimethyl chromone	96	MOL006635	oxysophocarpine
40	MOL006578	2-n-pentacosyl-5,7-dihydroxy-6,8-dimethyl chromone	97	MOL006637	Psi-ephedrin
41	MOL006579	2-n-pentadecyl-5,7-dihydroxy-6,8-dimethyl chromone	98	MOL006638	Pterocarpine
42	MOL006580	2-n-tricosyl-5,7-dihydroxy-6,8-dimethyl chromone	99	MOL006639	TNP00221

No.	Mol ID	Molecule Name	No.	Mol ID	Molecule Name
43	MOL006581	2-n-tridecyl-5,7-dihydroxy-6,8-dimethyl chromone	100	MOL006640	sokurarinone
44	MOL006582	5α,9α-dihydroxymatrine	101	MOL006641	sophoraflavanone,g
45	MOL006583	7,11-dehydromatrine	102	MOL006642	sophoraflavoside II
46	MOL006584	9alpha-hydroxy-7,11-dehydromatrine	103	MOL006643	sophoraflavosideⅢ_qt
47	MOL006585	Kushenol I	104	MOL006644	sophoraflavoside IV
48	MOL006586	Kushenol M	105	MOL006645	sophoraflavoside IV_qt
49	MOL006587	N-allomatrine	106	MOL006646	sophoraflavosideⅢ
50	MOL006588	N-oxysophocarpine	107	MOL006647	sophoraisoflavanone,a
51	MOL006589	Artepillin C	108	MOL006648	sophoranol N-oxide
52	MOL006590	Baptifoline	109	MOL006649	sophranol
53	MOL006591	Thc-9-cooh	110	MOL006650	(-)-Maackiain-3-O-glucosyl-6'-O-malonate
54	MOL006592	7-Demethylsuberosin	111	MOL006651	Trifolirhizin
55	MOL006593	Deoxyhumulone	112	MOL006652	trifolrhizin
56	MOL006594	Eciphin	113	MOL006653	xanthohumol
57	MOL006595	(2R)-flavanone			

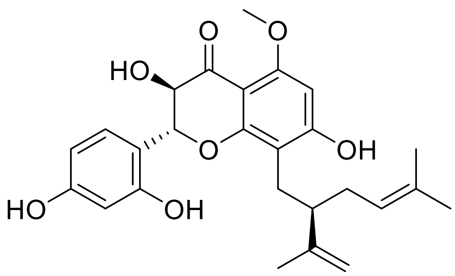

kushenol I

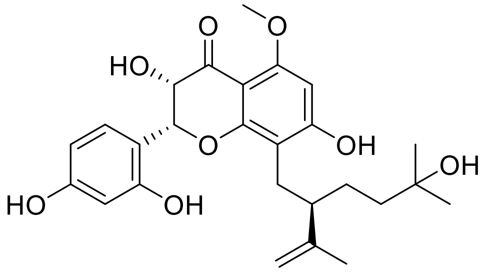

kushenol K

Fig. (S1). The 2D structures of kushenol I and kushenol K.
